# Medial prefrontal cortex: genes linked to bipolar disorder and schizophrenia have altered expression in the highly social maternal phenotype

**DOI:** 10.3389/fnbeh.2014.00110

**Published:** 2014-04-02

**Authors:** Brian E. Eisinger, Terri M. Driessen, Changjiu Zhao, Stephen C. Gammie

**Affiliations:** ^1^Department of Zoology, University of Wisconsin-MadisonMadison, WI, USA; ^2^Neuroscience Training Program, University of Wisconsin-MadisonMadison, WI, USA

**Keywords:** microarray, bipolar disorder, schizophrenia, maternal behavior, social behavior

## Abstract

The transition to motherhood involves CNS changes that modify sociability and affective state. However, these changes also put females at risk for post-partum depression and psychosis, which impairs parenting abilities and adversely affects children. Thus, changes in expression and interactions in a core subset of genes may be critical for emergence of a healthy maternal phenotype, but inappropriate changes of the same genes could put women at risk for post-partum disorders. This study evaluated microarray gene expression changes in medial prefrontal cortex (mPFC), a region implicated in both maternal behavior and psychiatric disorders. Post-partum mice were compared to virgin controls housed with females and isolated for identical durations. Using the Modular Single-set Enrichment Test (MSET), we found that the genetic landscape of maternal mPFC bears statistical similarity to gene databases associated with schizophrenia (5 of 5 sets) and bipolar disorder (BPD, 3 of 3 sets). In contrast to previous studies of maternal lateral septum (LS) and medial preoptic area (MPOA), enrichment of autism and depression-linked genes was not significant (2 of 9 sets, 0 of 4 sets). Among genes linked to multiple disorders were fatty acid binding protein 7 (*Fabp7*), glutamate metabotropic receptor 3 (*Grm3*), platelet derived growth factor, beta polypeptide (*Pdgfrb*), and nuclear receptor subfamily 1, group D, member 1 (*Nr1d1*). RT-qPCR confirmed these gene changes as well as FMS-like tyrosine kinase 1 (*Flt1*) and proenkephalin (*Penk*). Systems-level methods revealed involvement of developmental gene networks in establishing the maternal phenotype and indirectly suggested a role for numerous microRNAs and transcription factors in mediating expression changes. Together, this study suggests that a subset of genes involved in shaping the healthy maternal brain may also be dysregulated in mental health disorders and put females at risk for post-partum psychosis with aspects of schizophrenia and BPD.

## Introduction

The establishment of the maternal phenotype is one of the most dramatic biological transformations known in mature mammals. It involves changes across many physiological systems that prepare an individual to meet the demands of motherhood, including alterations within the brain that lead to the onset of adaptive parental and social behaviors (Lightman, 1992; Windle et al., 1997; Russell et al., 2001; Smith and Grove, 2002; Neumann, 2003; Slattery and Neumann, 2008; Levy et al., 2011). However, motherhood also carries a risk for post-partum depression, which affects 10–13% of new mothers. More debilitating “post-partum psychosis” afflicts 1–2 women per 1000 with rapid onset of symptoms in the first 2–4 weeks after delivery (Paffenbarger, 1964; Kendell et al., 1987). Post-partum psychosis has been described as an overt presentation of BPD, and to a lesser extent, schizophrenia, triggered in the post-partum period (Sit et al., 2006; Spinelli, 2009). It is therefore possible that naturally occurring changes which contribute to regulation of anxiety, emotional reactivity, and sociability in new mothers also contribute to similar processes in contexts of dysregulation, including pathological mood and personality disorders in humans (Lukas and Neumann, 2013). The post-partum mouse represents an ideal model system in which the genetics of the maternal brain can be explored with relevance to mental health. Maternal behavior is the product of numerous brain regions which together constitute an interconnected network (Slotnick, 1967; Slotnick and Nigrosh, 1975; Numan et al., 1977; Gammie et al., 2007; Lee and Gammie, 2009, 2010; Numan and Stolzenberg, 2009; Scotti et al., 2011). Recent experiments have begun to characterize large scale gene expression changes of lactating mice in various parts of this network, including septal regions and medial preoptic area (MPOA) (Zhao et al., 2012; Eisinger et al., 2013b; Driessen et al., 2014).

The medial prefrontal cortex (mPFC) is anatomically incorporated in maternal behavior circuitry. mPFC sends projections to many structures, including lateral septum (LS), nucleus accumbens (NA), and MPOA, and receives dopaminergic input from the ventral tegmental area (VTA) (Christie et al., 1985; Takagishi and Chiba, 1991; Sheehan et al., 2004; Olazabal et al., 2013). mPFC has been implicated in maternal behavior by a myriad of imaging studies in humans. fMRI has shown that mPFC in mothers exhibits significant activity in response to a variety of visual and audio infant stimuli (Lorberbaum et al., 2002; Noriuchi et al., 2008), which appears to be coordinated with a neural network that integrates cognitive and affective information in the production of motor and behavioral outputs (Wise, 2000; Strathearn et al., 2008). Human mPFC is one of several brain areas that increase in volume throughout the post-partum period, suggesting a possible role for long term structural changes in the maternal phenotype (Kim et al., 2010). In rats, mPFC lesions result in impaired pup retrieval and pup licking, but do not affect nest building (Afonso et al., 2007). Neural inactivation with bupivacaine infusion reveals that mPFC sub regions participate in motivation circuitry to mediate maternal behavior in the post-partum period (Pereira and Morrell, 2011). Additionally, mPFC has been implicated in numerous psychiatric disorders; PET scan experiments show that human patients suffering from schizophrenia have altered neurotransmitter production in mPFC compared to controls. Thus, mPFC is a critical component of both maternal care and aspects of mental health diseases.

In order to explore the genetic basis of mPFC's contribution to the maternal phenotype, which includes changes in emotional reactivity and sociability, we assessed large scale gene expression changes in the mPFC of post-partum, lactating mice relative to virgin control females. We employed a housing and pairing paradigm which is known to successfully yield animals that display a robust maternal behavioral phenotype and coupled it to an oligonucleotide microarray analysis. In addition to identifying genes with significantly altered expression in the maternal brain, results were also analyzed using multiple systems-level approaches, including NIH's DAVID functional annotation clustering tool, and a novel tool known as the Modular Single-set Enrichment Test (MSET) that tests enrichment of gene sets linked to numerous mental health disorders (Eisinger et al., 2013a). To explore the regulation of gene expression in the maternal brain, microRNAs, and transcription factors were considered. Enrichment of microRNA binding sites within microarray results was evaluated by ToppCluster. Weighted Gene Correlation Network Analysis (WGCNA) and the Animal Transcription Factor Database were used to identify a module of genes and transcription factors with highly concordant expression changes associated with the maternal phenotype.

## Materials and methods

### Animals

Mice used in this study were outbred hsd:ICR (Harlan, Madison WI) females ~70 days of age at the time of the experiment. All mice were nulliparous when obtained. Nulliparous animals that would become the “maternal” group were housed with breeder hsd:ICR males for a 2 weeks mating period, while those in the “virgin” control group were housed with age-matched female littermates to provide comparable levels of social interaction. While it has been shown that the effects of individual vs. group housing on stress levels in female mice can be variable among mouse strains (Arndt et al., 2009), multiple studies suggest that ICR males are healthier and less stressed when housed in groups (Ben-Nathan and Feuerstein, 1990; Huong et al., 2005; Kabuki et al., 2009), and group housed ICR females housed alone are more susceptible to virus-induced mortality (Ben-Nathan et al., 1991, 1995). After the mating period, all females from both groups were housed individually through parturition (post-partum day 0) until tissue collection (post-partum day 7). The housing and comparison strategy used in this study provides all subjects with similar levels of social interaction throughout the entirety of the experiment to help control for potential effects of housing on stress and gene expression. This paradigm has been used previously in our lab to examine gene expression changes associated with the collective events (pregnancy, parturition, and post-partum) that generate the maternal phenotype (Zhao et al., 2012; Eisinger et al., 2013b), and the maternal-virgin comparison is a proven approach for studying a wide variety of markers, including gene expression, that characterize the maternal phenotype (Mann et al., 1997; Neumann et al., 2000; Leuner et al., 2007; Kinsley and Amory-Meyer, 2011; Maeng and Shors, 2012; Shams et al., 2012). Subjects were provided with *ad libitum* access to breeder chow (Harlan, Madison WI) and water, and were housed with precut nesting material in polypropylene cages that were changed weekly prior to parturition, after which cages were not changed again until dissection. On day 0, litters were culled, if necessary, to standardize litter size to eleven. All subjects were kept on a 12:12 light:dark cycle with lights on at 6:00 CST. All procedures followed guidelines set by the National Institutes of Health Guide for the Care and use of Laboratory Animals, and were approved by the University of Wisconsin Animal Care and Use Committee.

### Tissue collection and RNA extraction

**Figure 1:**
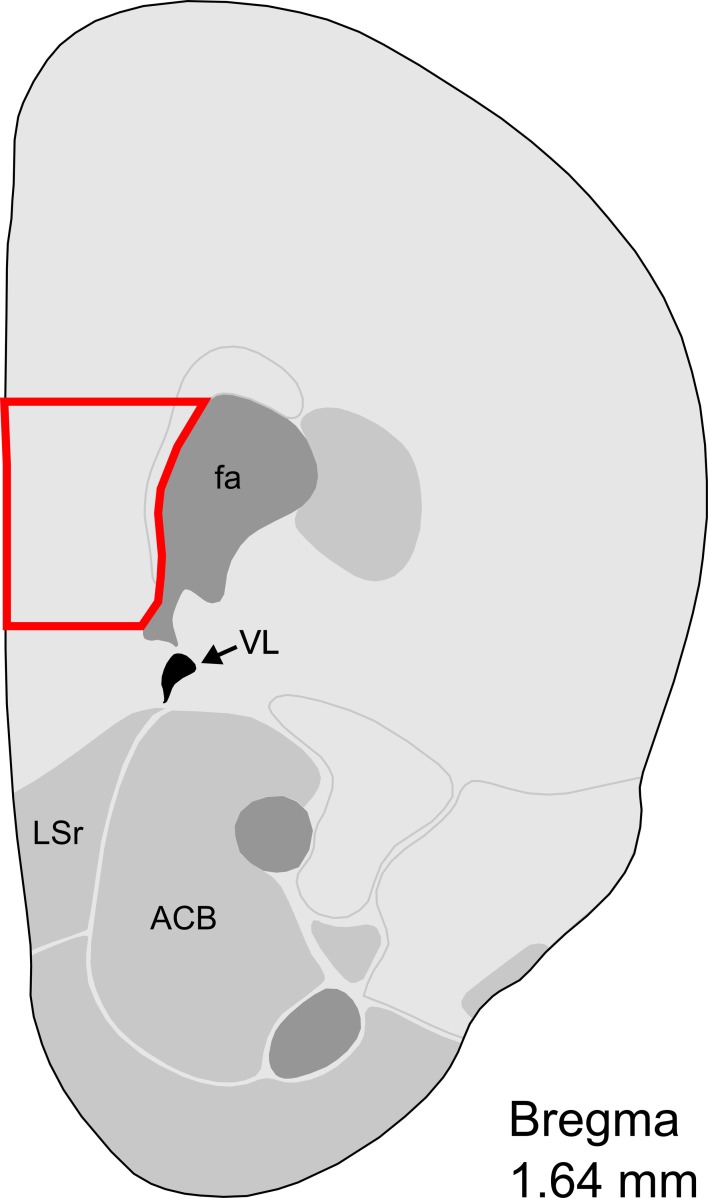
**Representative section with mPFC dissection for microarray analysis**. Distance from Bregma in the rostrocaudal plane is indicated. Modified from the Allen Mouse Brain Atlas (reference atlas version 1, 2008). Abbreviations: fa, corpus callosum, anterior forceps; VL, lateral ventricle; LSr, lateral septum, rostral; ACB, nucleus accumbens.

On post-partum day 7, when maternal behaviors are known to be highly and stably exhibited, virgin and post-partum females were lightly anesthetized with isuflurane and decapitated between 9:00 and 12:00 CST. Brains from age-matched virgin females were collected on the same day as their post-partum counterparts, and dissections were alternated between groups. After decapitation, vaginal lavage was performed on virgin subjects to determine estrous state. To control for possible effects of estrous cycling on gene expression, only diestrous virgins were included in the microarray experiment (Romano et al., 1988; Arosh et al., 2002). Brains were flash frozen in isopentane and stored at −80°C until being sectioned by cryostat (Leica CM1850, Bannockburn, IL, USA) at 200 micrometer thickness and mounted on gelatin-coated slides. mPFC was collected with a micropunch technique (Makino et al., 1994) and the Brain Punch Set (Stoelting, Wood Dale, IL, USA) under a dissecting microscope. mPFC tissue was collected from Bregma 1.98 to 1.54 mm in an area located medially from the anterior forceps of the corpus callosum to the midline, which included the prelimbic area, infralimbic area, and part of the anterior cingulate area (Figure 1). While mPFC subregions can contribute differentially to some aspects of behavior, we collected this inclusive area in accordance with previous studies of maternal behavior (Afonso et al., 2007; Pereira and Morrell, 2011) which found largely concordant and complementary effects of these mPFC regions on maternal responsiveness. Samples were collected from 10 post-partum females and 10 virgin females, and were subsequently stored at −80°C until RNA extraction. Total RNA was extracted with the Aurum Total RNA Fatty and Fibrous Tissue Kit (Bio-Rad, Hercules, CA, USA) in accordance with minor adjustments to the manufacturer's instructions. Briefly, two low-stringency washes were added prior to RNA elution, and total RNA was eluted with 30 μ L of nuclease-free water heated to 70°C, instead of the included elution solution. RNA concentration was measured with a NanoDrop 2000 spectrophotometer (Thermo Scientific, Wilmington, DE, USA) and stored at −80°C until further processing.

### High-density oligonucleotide array hybridization

Six samples from each group were randomly selected for inclusion in the microarray experiment. Microarray analysis was performed with the GeneChip Mouse Gene 2.0 ST Array (Affymetrix, Santa Clara, CA, USA) with targets derived from total RNA isolated from mPFC as described above. cDNA for array hybridization was reverse transcribed from 200 ng of total RNA with the Ambion GeneChip WT Expression Kit (Ambion, Austin, TX) according to manufacturer's instructions. In short, total RNA was used as template in the synthesis of double-stranded cDNA, which was then used to synthesize single-stranded cRNA. This cRNA was subsequently used as a template for one round of single-stranded cDNA synthesis, and the resulting DNA-RNA hybrids were degraded with RNase H. Amplified single-stranded cDNA was fragmented and biotinylated with an Affymetrix WT Terminal Labeling Kit (Affymetrix, Santa Clara, CA, USA) according to the manufacturer's specifications. Fragmented, labeled cDNA samples were hybridized with the arrays for 16 h at 45°C. Hybridized arrays were washed, stained, and scanned at 570 nm on an Affymetrix GC3000 G7 Scanner. During scanning, a fluid leak occurred in one chip and high regional background was detected in another (one maternal and one virgin), so analysis of results was performed with an N of 5 per group. Data were extracted and processed in the Affymetrix Command Console v. 3.1.1.1.229. cDNA synthesis, fragmentation, labeling, array hybridization, staining, and scanning were performed by the Gene Expression Center at the University of Wisconsin-Madison.

### Probeset level summarization and microarray statistical analysis

Probeset level summarization and normalization were performed using the probe logarithmic intensity error (PLIER) algorithm in Affymetrix Expression Console, build 1.2.1.20. The BioConductor package limma v3.14.4 was used to perform an array-specific empirical Bayesian implementation of ANOVA to generate inferential statistics of differential expression between genes in the virgin and post-partum mPFC samples. Nominal and false discovery rate (FDR) adjusted *p*-values were calculated. Because so few genes had FDR-adjusted *p*-values under the commonly used significance threshold of 0.25, the nominal PLIER *p*-value was used for all analyses. Fold change was calculated for each gene as the ratio of the limma-calculated average maternal expression coefficient divided by average virgin expression coefficient. Expression data have been uploaded to the NIH's Gene Expression Omnibus with accession number GSE54426.

### Functional enrichment with NIH's david functional annotation clustering

All microarray targets with *p*-values less than 0.01 were used as input for NIH's DAVID functional annotation clustering tool (Huang Da et al., 2009). Among these results, DAVID recognized 659 mouse gene IDs. Default annotation systems for functional categories, gene ontology, pathways, and protein domains were selected, and the default medium classification stringency was applied for clustering enriched gene sets. A cluster was considered to be enriched if its enrichment score was greater than 1.3, which corresponds to a geometric *p*-value mean of 0.05 for the cluster's constituent gene sets.

### Modular single-set enrichment test (MSET)

MSET was used to test significant microarray results (*p* < 0.02) for enrichment of gene lists associated with autism, schizophrenia, and BPD. These gene sets were extracted from online gene-disease association databases, including AutDB by the non-profit organization Mindspec, the Autism Genetic Database (AGD) (Basu et al., 2009), the HuGE Navigator's Phenopedia, the DISEASES database by the Novo Nordisk Foundation Center for Protein Research at the University of Copenhagen (http://diseases.jensenlab.org/Search), the NIH's Genetic Association Database (GAD) (Becker et al., 2004), the Weizmann Institute of Science's MalaCards compendium (Rappaport, 2013), and the Schizophrenia Research Forum's SZGene database (Allen et al., 2008). Additional candidate autism gene sets were taken from two recent publications; one utilizing a noise-reduction genome wide association study method (Hussman et al., 2011), and one using a “seeding” bioinformatics technique to build a predictive gene map for the disease (Kumar et al., 2011).

MSET uses a randomization testing algorithm to assess overrepresentation of disease-associated genes within significant microarray results. It generates a null distribution of expected number of matches to a given database of interest in randomly generated results built by sampling without replacement from the entire microarray background (Eisinger et al., 2013a). A *p*-value is then derived from the proportion of randomly generated results that included at least as many matches to the database as those found in the actual significant results from the microarray experiment. In this study, we tested enrichment within microarray results with *p*-values less than 0.02 (*n* = 1204 annotated genes) using 10,000 randomized sets of results. While *p* = 0.01 was used as a threshold in other systems-level analyses performed, *p* = 0.02 was chosen for this application because a larger set of results allows for greater number of matches to database in randomized results, which is important for generating a null distribution that is normally distributed with a broad enough range to allow accurate hypothesis testing. Further, current and previous evaluations indicate that almost all of the genes in this range can be confirmed via qPCR and thus are biologically relevant (Saul et al., 2012; Eisinger et al., 2013b). Gene sets associated with ADHD were tested, but were not included in the analysis because their respective null distributions were too narrow and too discrete to be reliably interpreted.

### MicroRNA enrichment analysis with ToppCluster

ToppCluster recognized 585 gene symbols amongst microarray targets with *p*-values less than 0.01 (Kaimal et al., 2010). The default statistical correction and threshold of Bonferroni-adjusted *p*-value less than 0.05 was applied to microRNA enrichment analysis. Two analyses were conducted in parallel using two different input gene lists—one consisting of genes that were upregulated in the maternal group (*n* = 330), and one of downregulated genes (*n* = 355). Sequences for microRNAs that reported enrichment for binding sites in the resulting output tables were extracted from miRBase (http://www.mirbase.org/), and duplicate microRNAs predicted by multiple prediction systems aggregated by ToppCluster were removed such that each microRNA was represented only once.

### WGCNA and transcription factor analysis

WGCNA was used to identify modules of genes whose expression changes are highly correlated to one another in the maternal mPFC compared to virgin within results with *p*-values under 0.01 (*n* = 824 probes). The free statistical software R was used for all WGCNA computations (Zhang and Horvath, 2005; Langfelder and Horvath, 2008). To generate a weighted network of genes (nodes) and their expression correlations (edges), correlations were raised to a soft thresholding power β, chosen such that the network approximates a model of scale-free topology (*R*^2^ > 0.8), which is a necessary assumption for WGCNA accuracy. Using unsupervised hierarchical clustering, a minimum module size of 50 genes, and a threshold setting for merging modules of 0.25, WGCNA identified one module of 204 genes that was positively correlated to the maternal sample group. This module was exported as a Cytoscape network file, which was manually trimmed to consist only of transcription factor nodes and their gene-to-gene correlations. The finalized transcription factor module was visualized with Cytoscape v3.0.1.

### Quantitative real-time PCR

To confirm expression changes detected by microarray analysis, qPCR was performed on genes of interest (*n* = 10 per group). Target genes were *Pdgfrb*, *Grm3*, *Flt1*, *Penk*, and *Nr1d1*. Two stable reference genes were used to normalize relative expression results of genes of interest; Tyrosine 3-monooxygenase/tryptophan 5-monooxygenase activation protein, zeta polypeptide (*Ywhaz*), and peptidylprolyl isomerase A (*Ppia*). Primer information can be viewed in Supplementary Table 2.

A SuperScript III First-Strand Synthesis System for RT-PCR (Invitrogen, Carlsbad, CA, USA) was used to reverse transcribe 100 ng of RNA to cDNA in an Eppendorf MasterCycler Personal PCR Machine (Eppendorf, Hamburg, Germany) with poly-T 20mer primers. The thermal profile used is as follows: an initial melting step of 95°C for 30 s, followed by 40 cycles of a 5-s 95°C melt, a 20-s 58°C annealing step, and a 20-s 72°C elongation step. A melt curve was performed from 60–95°C at 5-s 0.5°C increments to confirm specificity of primer binding, and relative expression values were calculated with REST 2009.

## Results

### Genes with altered expression in mPFC

High density oligonucleotide microarray was performed on mPFC tissue collected from virgin and post-partum animals (Figure 1), and results were analyzed with the Probe Logarithmic Intensity Error (PLIER) algorithm. Of the 41,346 probes on the platform, 824 probes representing 685 unique, annotated genes reported altered expression in maternal mPFC compared to virgin with *p*-values less than 0.01. A summary of the full set of results is available in Supplementary Table 1, and the complete expression data is available on the Gene Expression Omnibus with accession number GSE54426.

**Figure 2:**
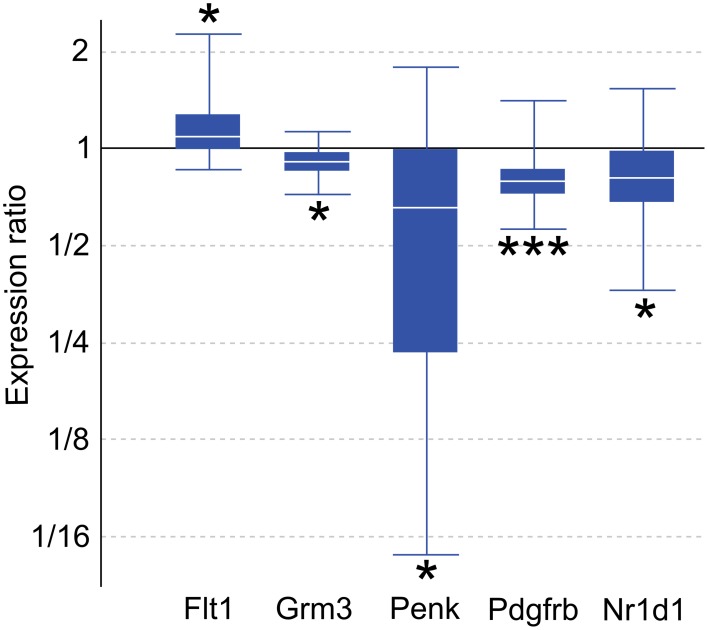
**Quantitative real-time PCR confirmation of expression changes for genes of interest in maternal mPFC compared to virgin**. Relative expression distribution (Y-axis) represented as a ratio of lactating maternal vs. virgin (*n* = 10 per group) normalized against two references genes, *Ppia* and *Ywhaz*, and shown by box-and-whisker plots as medians (white lines), interquartile ranges (boxes), and ranges (whiskers). Ratios over one indicate genes that are more highly expressed in lactating maternal mPFC than in virgin. ^*^p < 0.05; ^***^p < 0.001.

Genes with altered expression in maternal mPFC span many functional categories, including metabolic processes, regulation of transcription, development, transport, and neurotransmission. Genes of interest for quantitative real-time PCR (qPCR) confirmation were selected based on biological importance, statistical significance in the present microarray, and concordance with recent and ongoing expression studies in various other brain regions associated with maternal behavior. Significant targets in the microarray results (*p* < 0.05) include the platelet derived growth factor, beta polypeptide (*Pdgfrb*), glutamate metabotropic receptor 3 (*Grm3*), and FMS-like tyrosine kinase 1 (*Flt1*). Several genes of interest displayed robust expression changes in the maternal mPFC with borderline significance as reported by microarray analysis, including proenkephalin (*Penk*), and nuclear receptor subfamily 1, group D, member 1 (*Nr1d1*). qPCR experiments confirmed expression changes for all genes of interest (Figure 2) with the following maternal/virgin expression ratios (>1.0 indicating upregulation in post-partum state, <1.0 reflecting downregulation): *Pdgfrb* (*p* = 0.001, fold change 0.81), *Grm3* (*p* = 0.018, fold change 0.92), *Flt1* (*p* = 0.040, fold change 1.15), *Penk* (*p* = 0.032, fold change 0.50), and *Nr1d1* (*p* = 0.031, fold change 0.79). qPCR carried out for fatty acid binding protein 7, brain (*Fabp7*) and plasma membrane proteolipid (*Pllp*) in our mPFC samples for a separate, cross-regional expression study confirmed significantly reduced expression of both genes and further validated the microarray technique (Driessen et al., 2014).

### Functional profiling of genes with significant expression changes in maternal mPFC

**Figure 3:**
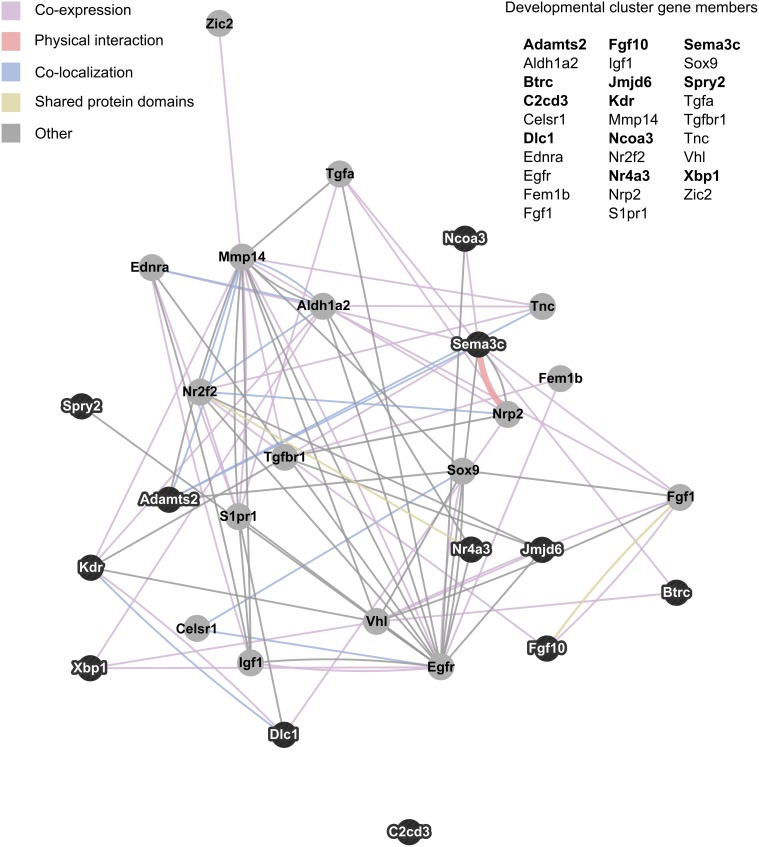
**Interaction network of developmentally related genes with altered expression in maternal mPFC**. The 29 genes that appeared in two or more developmental clusters recognized by NIH's DAVID functional annotation clustering as enriched within significant maternal mPFC expression results were visualized as an interaction network in GeneMania. Gene symbols in bold text are upregulated in lactating maternal mPFC relative to virgin, and are represented in the network image as dark nodes with white text. Non-bold gene symbols and gray nodes with dark text correspond to genes that are downregulated. The nature of the interaction data linking any two nodes is encoded by color, and distance between nodes is proportional to the strength of evidence for their interaction.

NIH's DAVID functional annotation clustering tool was used to assess enrichment of functionally related groups of genes within the significant results (*p* < 0.01). With a medium classification stringency setting, 14 clusters of genes were found to be enriched. Of these, the most prevalent biological pathways represented were developmental in nature. Six clusters represented developmental processes, including cell migration/motility, and morphogenesis of tissue and glands. The 29 genes that appeared in two or more developmentally-related clusters were consolidated into a single group and visualized as an interaction network with GeneMania (Figure 3).

### MSET analysis for enrichment of gene sets associated with mental health disorders

MSET, a randomization testing script, was used to assess enrichment for genes associated with autism, BPD, depression, and schizophrenia within genes displaying altered expression (*p* < 0.02) in maternal mPFC on the microarray platform. Disease-linked gene modules were extracted from 10 independent sources, including databases which curate gene-disease associations for multiple diseases as well as those that are specific to a particular disease. Two autism-associated gene modules were pulled from recent publications that employ novel techniques to generate original candidate autism gene lists.

**Figure 4:**
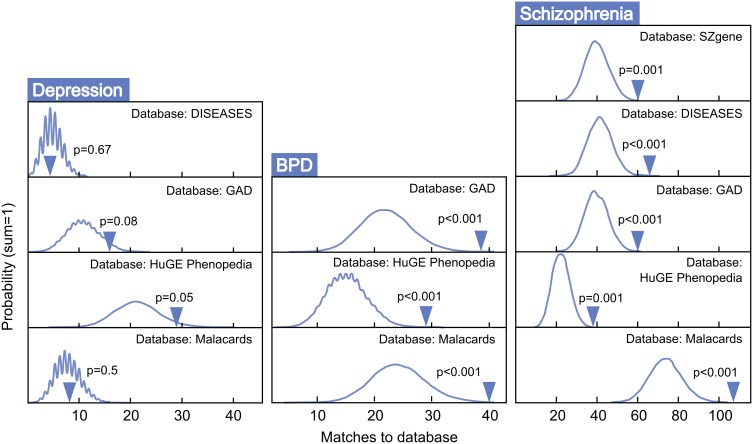
**MSET evaluation of enrichment for depression, BPD, and schizophrenia-associated gene sets within significantly altered genes in maternal mPFC**. Y-axis represents the probability of X matches to database appearing in a randomly generated set of simulated results from the microarray background. The blue arrow shows how many matches were found in the actual significant maternal mPFC expression changes and where that number falls on the probability density distribution. The enrichment *p*-value is derived from the number of simulated results that contained at least as many matches to database as the actual results.

Figure 4 presents MSET analysis for gene sets linked to BPD, schizophrenia, and depression. Genes related to BPD were highly enriched, with three of three databases reporting *p*-values under 0.01. Similarly, all five schizophrenia-associated gene databases reported enrichment *p*-values under 0.01. In contrast, only two of four depression databases exhibited borderline significance, with an average *p*-value of 0.33 across all four. Two out of nine autism-related gene sets tested showed enrichment (*p* < 0.05), with a non-significant mean *p*-value of 0.34 for all nine together (data not shown). As a negative control, enrichment for arthritis-associated gene sets was also assessed and proved to be non-significant with an average *p*-value of 0.49 across five modules tested.

Genes that were differentially regulated in maternal mPFC as reported by microarray analysis (*p* < 0.02) that appeared in all databases tested for BPD and schizophrenia are listed in Table 1 as particularly strong candidate genes for mental health disorders at work in the maternal brain.

**Table 1:** **Genes with strong disease-associations that exhibit altered expression in maternal mPFC**.

**BPD**							
*Chrm2*	*Cry2*	*Csnk1d*	*Csnk1e*	*Fabp5*	*Fabp7*	*Fyn*	*Grm3*
*Hspa5*	*Ncam1*	*Nos1ap*	*Npas3*	*Nr4a2*	*Per3*	*Sorcs2*	*Syne1*
*Xbp1*							
**SCHIZOPHRENIA**							
*Chrna4*	*Cplx1*	*Csnk1e*	*Dlg1*	*Fabp7*	*Fgfr1*	*Fyn*	*Grik3*
*Grm3*	*Grm5*	*Grm7*	*Homer1*	*Ncam1*	*Nos1ap*	*Npas3*	*Nr4a2*
*Pdyn*	*Per3*	*Plxna2*	*Ptprz1*	*Slc25a27*	*Xbp1*		

Each gene listed for a particular disorder is featured in all gene-disease association databases tested in this study for that disorder.

### Roles for microRNAs and transcription factors in altered expression of genes in maternal mPFC

Because the RNA purification technique used in this experiment did not allow for direct analysis of microRNA in the microarray data, a computational method was applied. ToppCluster revealed that there was striking enrichment of predicted microRNA binding sites within significantly altered genes in maternal mPFC. With the default threshold setting of 0.05 for Bonferroni-adjusted *p*-values, ToppCluster analysis found enrichment of 36 miRNA binding sites within genes that were upregulated in the maternal brain (*n* = 330), and 8 in genes that were downregulated (*n* = 355) (Table 2). No significant enrichment was detected for transcription factors within significant results by ToppCluster, although it was observed that significant microarray results did include 40 transcription factors, 4 chromatin remodeling factors, and 13 cofactors as listed in the Animal Transcription Factor Database.

**Table 2:** **Enriched miRNA binding sites in genes that are upregulated and downregulated in maternal mPFC**.

**Upregulated**			**Downregulated**
miR-103	miR-200c	miR-302d	miR-124a
miR-106a	miR-204	miR-302e	miR-340
miR-106b	miR-20a	miR-372	miR-374a
miR-107	miR-20b	miR-373	miR-374b
miR-135a	miR-211	miR-429	miR-485-3p
miR-135b	miR-218	miR-519d	miR-562
miR-138	miR-29a	miR-520a-3p	miR-606
miR-141	miR29b	miR-520b	miR-607
miR-15b	miR-29c	miR-520c-3p	
miR-17	miR-302a	miR-520d-3p	
miR-182	miR-302b	miR520e	
miR-200b	miR-302c	miR-93	

**Figure 5:**
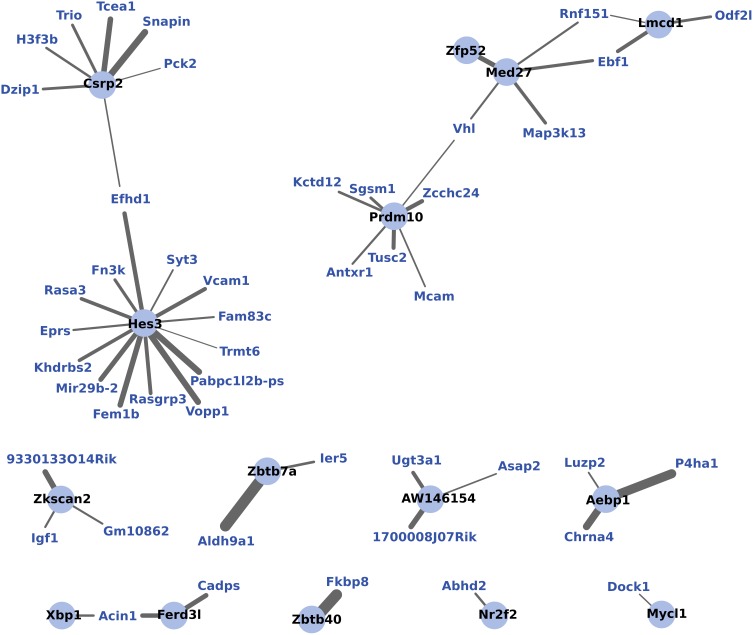
**WGCNA network of transcription factors and genes with highly correlated expression changes in maternal mPFC**. Within the gene module identified by WGCNA as having highly correlated expression, transcription factors (light blue circles with black labels) are visualized in relation to other module genes (blue labels). The significance of the correlation is indicated by the width of the line, with thicker lines reflecting more significant correlation.

WGCNA identified one module of 204 genes whose expression changes were correlated to the maternal phenotype (*R*^2^ = 0.93, *p* < 0.01) and were also strongly correlated to one another within the module (*R*^2^ = 0.77, *p* < 0.01). NIH's DAVID functional annotation clustering failed to show a predominant functional enrichment for this group (data not shown). To investigate a possible role for transcription factors in the coordinated expression of this gene network, expression correlations for 15 transcription factors found within this module were visualized in Cytoscape (Figure 5).

## Discussion

This study used a microarray approach to evaluate naturally occurring large scale gene expression changes in the mPFC of lactating, maternal mice compared to nulliparous controls. The resulting expression patterns are not necessarily a direct cause or result of maternal behavior *per se*, but are correlated with the healthy maternal phenotype, which itself is shaped by a variety of contributing social and biological factors. In addition to confirming differential expression of several genes that may be important markers of the maternal phenotype, systems level analyses detected enrichment for developmental processes and genes related to schizophrenia and BPD within significant results. Enrichment for miRNA binding sites suggests that transcriptional regulation via miRNA mechanisms could be influential in shaping the maternal brain.

### Expression changes in maternal mPFC are enriched for genes linked to bipolar disorder and schizophrenia, but not autism or depression

MSET is a randomization testing procedure that assesses the degree to which disease-linked genes are overrepresented in a set of significant microarray results compared to what would be expected by chance. The maternal mPFC microarray results (*p* < 0.02) showed little enrichment for autism-associated gene sets (two of nine databases tested, average *p* = 0.34, data not shown), which is of interest because it differs from the compelling autism enrichment found in the gene expression profile of maternal LS (Eisinger et al., 2013a) and MPOA (Driessen et al., 2014). It should be noted that the two autism-linked gene sets that showed enrichment in maternal mPFC differed from the other seven in two ways; firstly, that they are original candidate autism gene lists generated by recent publications, while the others are databases of associations aggregated from a large volume of scientific literature (Hussman et al., 2011; Kumar et al., 2011). Secondly, they share very little similarity in constituent genes (~9%) with other autism lists used in the present study.

Post-partum psychosis develops suddenly in the days and weeks following parturition, even in women with no history of mental illness, strongly suggesting that changes which take place in the transition to motherhood can put individuals at risk for mental health disorders. Human studies have proposed that, along with broadly-defined schizophrenia, post-partum psychosis is comprised predominantly of BPD with acute onset and phasic symptomatology (Da Silva and Johnstone, 1981; Pfuhlmann et al., 1998). Striking enrichment was detected within expression changes in the present study for both schizophrenia and BPD (*p* < 0.01, Figure 4), indicating that gene changes identified in the mPFC of new mothers may be putting women at risk for post-partum psychosis with BPD and schizophrenia-like pathologies. Genes at work in maternal mPFC with strong links to BPD and schizophrenia (Table 1) may represent a core basis for these diseases in non-maternal contexts as well—future MSET analysis of additional animal models and human data could reinforce this hypothesis.

Surprisingly, maternal mPFC was not enriched for depression-linked genes (Figure 4). One possibility is that post-partum depression may be an etiologically distinct phenomenon from post-partum psychosis and is mediated by gene changes in areas other than mPFC. Alternatively, genes linked to general depression from association experiments may not reflect the true biology of depression occurring in the post-partum state, and post-partum depression symptoms could emerge from a BPD-like genetic basis. Despite the lack of compelling enrichment in depression-linked genes, MSET identified corticotropin releasing hormone (CRH) binding protein (*Crhbp*) as the lone gene dynamically regulated in maternal mPFC that appeared in all depression databases utilized in this study. Human genetic studies implicate the CRH pathway in mediating anxiety and depression symptoms (Binder and Nemeroff, 2010), and knockout studies have shown that *Crhbp* deletion impairs aspects of maternal care in mice (Gammie et al., 2008). It was also of interest that MSET identified circadian rhythm genes including period circadian clock 3 (*Per3*) and cryptochrome 2 (*Cry2*) in maternal mPFC with multiple links to depression, as clock genes are emerging as core contributors to depressive pathology in humans and animal models (Bunney and Bunney, 2000). Additional circadian genes are discussed below.

While maternal LS previously showed enrichment for schizophrenia and BPD, the genes that contributed to enrichment of each disease differed notably from those in mPFC (Eisinger et al., 2013a). For example, *Grm3* appears in all five schizophrenia gene lists extracted, presumably reflecting its strong association with the disorder in past studies. It is highly significant in both LS and mPFC arrays, while other glutamate-related genes strongly linked to schizophrenia, such as the kainate receptor 3 (*Grik3*), and metabotropic glutamate receptors 5 and 7 (*Grm5*, and *Grm7*), are only significant in mPFC (Table 1). This highlights the fact that different brain structures that comprise the maternal behavior circuit have unique genetic “signatures” at two levels; which diseases are enriched, and what individual genes account for enrichment. It is likely that this reflects the functional differences of each region and their contributions to disease symptoms. These findings can inform future efforts to target particular disorders with drugs that act only on regions and genes that contribute to their pathology. In addition, disease-linked genes that have consistently altered expression in the maternal brain across numerous regions, such as *Fabp7*, are of interest because of their widespread actions. *Fabp7* appeared in every BPD and schizophrenia-associated gene set tested in this study (Table 1).

### Genes of interest in maternal mPFC

Four genes of interest, *Grm3*, *Penk*, *Pdgfrb*, and *Nr1d1*, were found to be downregulated in maternal mPFC by qPCR analysis, while *Flt1* was upregulated (Figure 2). As noted previously, *Fabp7* was confirmed as part of a separate study.

*Grm3* is a member of the metabotropic glutamate receptor family. Glutamate activity in PFC has been associated with schizophrenia in numerous human studies (Ben-Nathan et al., 1991, 1995; Neumann et al., 2000), and converging lines of evidence suggest that *Grm3* in particular influences glutamate neurotransmission, PFC function, and risk for schizophrenia (Mann et al., 1997; Kinsley and Amory-Meyer, 2011). A selective agonist for group II metabotropic glutamate receptors (*Grm2* and *Grm3*) has proven to be an effective anti-psychotic treatment for schizophrenic patients in clinical trials (Shams et al., 2012). While *Grm3* has not yet been investigated in human mothers, the 8% decrease in *Grm3* mRNA measured by qPCR in the present study suggests that the glutamatergic pathway in which it acts may be an important influence on maternal behavior. Additional microarray results implicating glutamate transmission in maternal mPFC (*p* < 0.05) include *Grm5*, *Grm7*, *Grik3*, an aspartate/glutamate transporter (*Slc1a6*), and glutamate receptor interacting proteins 1 and 2 (*Grip1*, *Grip2*, Supplementary Table 1).

Opioid signaling, particularly via the μ-opioid receptor, is well-known to mediate analgesic, euphoric, and addictive effects upon the binding of exogenous ligands such as morphine (Neumann et al., 2000). The related δ-opioid receptor also participates in pain-reducing pathways, and knockout studies demonstrate that mice deficient for μ - and δ-opioid receptors exhibit opposing emotional phenotypes, in which *Oprd1*^−/−^ animals have elevated anxiety (Filliol et al., 2000). Knocking out *Penk*, the gene encoding the peptide precursor to the endogenous enkephalin ligands of the δ-opioid receptor, generates a similar phenotype of heightened anxiety in behavioral experiments (Ragnauth et al., 2001; Pradhan et al., 2011; Maeng and Shors, 2012). Our qPCR results show a significant, dramatic downregulation of *Penk* mRNA by nearly 50% in the maternal mPFC (Figure 2). The microarray data show that *Oprm1* is also differentially regulated in the maternal group (*p* = 0.031, Supplementary Table 1). It is likely that the involvement of the opioid system in the maternal brain is complex and could be involved in multiple maternal processes, including the bonding of mothers to offspring (Panksepp et al., 1994; Nelson and Panksepp, 1998).

*Pdgfrb* mRNA is reduced in the maternal PFC (Figure 2). *Pdgfrb* is a growth factor receptor that binds platelet-derived growth factors with B and D chains, and is involved in mitogenic pathways and cytoskeletal reorganization (Bergsten et al., 2001; Kaminski et al., 2001). It is essential for normal development of embryonic blood vessels in mice. *Flt1* is another gene related to vascular development that had altered expression in maternal mPFC and encodes the vascular endothelial growth factor receptor 1 (VEGFR1, Figure 2). While atypical cerebral blood flow in the PFC occurs in some schizophrenic patients, whether any of these blood vessel-related genes play a role in the disease is not known. In addition to its well-established role in angiogenesis in contexts of embryonic development and brain tumors, recent experiments have demonstrated that VEGFR1's ligand, VEGF, also has effects on neurons and glia that are relevant to neurological disorders (Carmeliet and Storkebaum, 2002). Cultured neurons from the peripheral nervous system treated with VEGF displayed increased axonal outgrowth and survival, while proliferation of Schwann cells was also observed (Sondell et al., 1999). Collectively, the altered expression of genes that influence the development of brain vasculature in maternal mPFC suggests that activation of latent differentiation and proliferation programs influencing both neurons and non-neuronal cells in the adult brain may underlie physiological changes in the maternal brain.

*Nr1d1* encodes a nuclear receptor transcription factor that interacts with clock proteins to regulate circadian rhythms. Circadian rhythms have been strongly linked to mood disorders—patients who suffer from depression, schizophrenia, and BPD commonly exhibit disturbances in sleep/wake cycles, appetite, and social rhythms (Boivin, 2000; Bunney and Bunney, 2000). It has been shown that the effects of lithium, the primary treatment for BPD, are mediated through activity of the kinase GSK-3β and *Nr1d1* (Yin et al., 2006). This biological pathway appears to be altered in the maternal mPFC, as qPCR reported a 21% reduction in *Nr1d1* transcripts compared to virgin animals (Figure 2). In addition, all three period genes (*Per1-3*) show upregulation in maternal mPFC in our microarray data (*p* < 0.05, Supplementary Table 1). Together, these preliminary results point to an involvement of circadian molecular circuitry within mPFC in regulating behavior in maternal animals.

### Enrichment of developmental processes within differentially regulated genes in maternal mPFC

A large proportion of gene clusters (6/14) found to be enriched in maternal mPFC microarray results by NIH's DAVID functional annotation clustering were primarily developmental in nature. These clusters represented biological pathways including cell migration/motility, blood vessel development, morphogenesis of tissue/glands, neural crest cell development, and respiratory development. While the relevance to mPFC of genes linked to respiratory development may be unclear, it is common for key developmental signaling genes to play a role in a variety of tissues (Barak et al., 1999; Reya et al., 2001; Reya and Clevers, 2005).

The transition to motherhood is sometimes described psychologically as a developmental stage akin to childhood, adolescence, and sexual maturity (Shectman, 1980). If the maternal brain does indeed represent a developmental endpoint, one would expect to see this notion recapitulated in systems-level modulation of genes involved with such processes in the brain. The enrichment of pathways related to development in maternal mPFC adds to a growing body of evidence that this is the case, in concordance with studies undertaken in maternal LS and MPOA (Eisinger et al., 2013b; Driessen et al., 2014). These genetic data in turn support anatomical and histochemical studies that demonstrate altered neurogenesis in the CNS of lactating females (Leuner et al., 2007; Pawluski et al., 2009). It is therefore likely that specific brain regions in the maternal mouse, including mPFC, undergo long term structural changes that support the emergence of maternal behavior in the lactating state.

Examining the relationships between developmentally-related genes with altered expression in maternal mPFC can also help generate hypotheses about specific pathways that may act in sculpting the maternal brain. Figure 3 maps these genes based on interaction data compiled from scientific literature. In this cluster, several genes stand out as particularly important due to high interconnectivity; epidermal growth factor receptor (*Egfr*), the aldehyde dehydrogenase (*Aldh1a2*), and matrix metallopeptidase 14 (*Mmp14*). The most extensively interconnected gene in Figure 3 is *Egfr*. This is not entirely surprising, as *Egfr* is a relatively well-studied gene for its central role in cell proliferation in many organisms, tissues, and in cancer (Yarden, 2001; Cheng et al., 2010). That it features so prominently in this cluster may be somewhat of an artifact due to its overrepresentation in the literature compared to other gene cluster members. *Aldh1a2* catalyzes the synthesis of retinoic acid, a hormonal signaling molecule that functions in developing and adult tissue (Everts et al., 2004; Vernet et al., 2006). Interestingly, *Aldh1a2* was identified as a candidate neuroprotective gene with increased expression in the adult mouse brain following hypoxia (Tang et al., 2006). *Mmp14* is involved with the breakdown of extracellular matrix in cell migration, and has been shown to be important for neurite outgrowth in mouse cerebellar neurons (Loers et al., 2012). A physical interaction visible in Figure 3 that could be important for development of the maternal mPFC is that of the receptor neuropilin 2 (*Nrp2*) and its semaphorin ligand, *Sema3c*. Experiments on *Nrp2* mutant mice reveal that this signaling system is crucial for axon guidance in cranial nerves, spinal nerves, and hippocampal mossy fibers (Chen et al., 2000; Giger et al., 2000). These genes and their associated pathways may represent promising targets for future studies of neural development in maternal brain tissue based on their conspicuous positions in the genetic network identified in maternal mPFC.

### MicroRNAs and some transcription factors may play a role in the positive regulation of transcription in the maternal brain

A previous study using Gene Set Enrichment Analysis (GSEA) on mouse septal regions unexpectedly found that a large number of microRNAs were linked to genes upregulated in the post-partum period, but not to downregulated genes (Zhao et al., 2012). The present microarray analysis used a different strategy on a different brain region, as we employed ToppCluster to calculate enrichment for predicted microRNA binding sites within differentially regulated genes in the maternal mPFC. With this method, evidence of microRNA involvement was again directionally preferential, with 36 binding sites found to be enriched in upregulated maternal genes, and only 8 in downregulated genes (Table 2).

One consideration to be made when interpreting these findings is that the bioinformatics services aggregated by ToppCluster (PITA, PicTar, TargetScan) operate on binding sites predicted by their algorithms, rather than binding sites established empirically. However, microRNA target prediction software has proven to be powerful thus far, as predicted sites in mammals have been reliably validated in numerous experiments (Lewis et al., 2003; Krek et al., 2005).

MicroRNAs were first characterized in *C. elegans* as short, double-stranded RNAs that mediate gene silencing by binding to the 3'UTR of target mRNA (Lagos-Quintana et al., 2001; Lau et al., 2001; Lee and Ambros, 2001). It is generally thought that microRNAs reduce gene expression, but experiments with cultured cells reveal that downregulation of microRNAs themselves can upregulate their target genes (Fontana et al., 2007), and others have shown that microRNAs may be able to switch from a repressing to activating function under certain conditions (Vasudevan et al., 2007). While elucidating the mechanism of microRNA action in maternal mPFC is beyond the scope of the current microarray experiment, the computational findings presented here add compelling evidence to the idea that they play a crucial role in regulating expression of genes in the post-partum, lactating brain.

To further investigate the coordination of gene expression in maternal mPFC, WGCNA analysis was used. WGCNA detects modules of genes whose expression changes are highly correlated to one another as well as to the experimental effect being tested, which is motherhood in this experiment. WGCNA detected a single module of 204 genes which featured 15 transcription factors. Figure 5 shows these transcription factors and the strength of their correlations to other genes within the gene module. It can be seen from this figure that the transcription factor gene *Hes3* is centrally positioned with extensive intra-modular correlations. *Hes3* is part of the notch signaling pathway, which is involved with neurogenesis (Androutsellis-Theotokis et al., 2006). Evidence has shown that *Hes3* plays a role in early development of the midbrain and hindbrain (Lobe, 1997; Hirata et al., 2001). Therefore, the finding that *Hes3* expression is centrally featured in a correlated network of expression changes in the maternal mPFC suggests that it may also be regulating developmental processes in the transition to motherhood.

### Methodological considerations

The maternal brain is shaped by numerous factors, including the experience of mating, the hormonal dynamics of pregnancy, parturition, lactation, and sensory input from offspring. These elements interact to generate gene expression changes in the post-partum state. The present study assesses gene expression associated with the healthy maternal phenotype, in which mothers show no signs of neglect or deficits in caring for offspring (zero mortality). Post-partum, lactating animals that have been exposed to the totality of these events were compared to naïve controls that have undergone none of them. Consequently, the contribution of each individual factor on expression patterns cannot be distinguished. The experimental paradigm utilized presently includes those experiential components which are most intrinsic to motherhood (mating, pregnancy, parturition, presence of pups), controls for those which are tractable (number of previous births, level of social interaction), and minimizes those which are least natural (housing-induced stress) to generate an ethologically relevant comparison. This approach has the advantage of allowing an understanding of the full extent of the multitude of gene expression changes occurring in mPFC in the maternal brain, but it also has limitations as no specific factor can be linked to a given change. However, the role of any of the given factors in contributing to gene expression changes can be evaluated in follow-up studies. Comparing lactating rodents to virgins has proven to be a valuable approach in past studies of maternal biology (Spinolo and Crowley, 1993; Miller et al., 2010; Jurek et al., 2012), and continuity in several consistent gene changes, such as *Fabp7*, across studies that differ in species, brain region, and group housing conditions (Xiao et al., 2005) suggests that such an approach is able to successfully identify a robust maternal phenotype. We acknowledge that minor effects of housing-induced stress on gene expression cannot be excluded, but this experimental design optimally reduces the possibility of this effect manifesting differentially between groups and confounding results. Collectively, the results presented in this study reflect the maternal phenotype itself, rather than the etiology of its components.

## Concluding remarks

In this study, we discovered that expression changes in mPFC of the highly social maternal mouse are highly enriched for genes associated specifically to BPD and schizophrenia, the symptoms of which have been clinically identified in the presentation of post-partum psychosis. Further, we identified a core subset of disease-linked genes that account for this enrichment. This does not indicate that the maternal brain itself is similar to a psychotic state—rather, we propose that genes with altered expression in the post-partum brain may have relevance to psychosis because if they are over-expressed or under-expressed relative to a normal maternal profile, this could be a source of the disorder. Genes which are naturally modulated in the transition to motherhood are the moving parts of sociability—subtle defects in expression levels of genes central to sociability and emotional reactivity are more likely to occur and have a pathological consequence than rarer gain-of-function or loss-of-function mutations in genes which are not naturally involved in the establishment of the maternal phenotype. The genetic network we have identified presently may therefore represent a shared basis for sociability and emotional reactivity in mPFC when appropriately modulated in the healthy maternal phenotype and when dysregulated in mental health disorders. This network is functionally characterized by developmental processes, indicating that behavioral changes in the post-partum period may include long-term structural changes in mPFC architecture. Finally, computational methods reveal that microRNAs could be important in preferentially mediating positive gene expression in the maternal state. These findings provide valuable insight into the dynamic genetics of social behavior and support the utility of the maternal brain for studying mental health in humans.

### Conflict of interest statement

The authors declare that the research was conducted in the absence of any commercial or financial relationships that could be construed as a potential conflict of interest.
