# Supplementary material for: Medial prefrontal cortex: genes linked to bipolar disorder and schizophrenia have altered expression in the highly social maternal phenotype
Source: Front Behav Neurosci. 2014 Apr 2;8:110. doi: 10.3389/fnbeh.2014.00110 (PMC3980118; doi:10.3389/fnbeh.2014.00110)
Supplement: Supplementary file 2 [file DataSheet2.DOCX]

| Gene symbol | Gene title | NCBI accession number |  | Primer sequence (5’->3’) | Primer position |
| --- | --- | --- | --- | --- | --- |
| *Pdgfrb* | Platelet derived growth factor receptor, beta polypeptide | NM_001146268.1 | Forward | TCATGAAGCCAGCAAGAGTG | 4882-4901 |
|  |  |  | Reverse | GTGGTAATCCCGTCAGCATC | 5044-5025 |
| *Grm3* | Glutamate receptor, metabotropic 3 | NM_181850.2 | Forward | TGAGTGGTTTCGTGGTCTTG | 2790-2809 |
|  |  |  | Reverse | GAATATGTGGTCGCAGTTCC | 2925-2906 |
| *Flt1* | FMS-like tyrosine kinase 1 | NM_010228.3 | Forward | AGGACTAGACAAGTAGGAAAGGG | 5905-5927 |
|  |  |  | Reverse | TCTGGTAGCCTCTGTCTCATC | 6049-6029 |
| *Penk* | Preproenkephalin | NM_001002927.2 | Forward | AGAGAGCACCAACAATGACG | 824-843 |
|  |  |  | Reverse | TGCTTCATCTTCCAGTTGGG | 914-895 |
| *Nr1d1* | Nuclear receptor subfamily 1, group D, member 1 | NM_145434.3 | Forward | GCTTCTCTCAGTTCCCACAAC | 1291-1311 |
|  |  |  | Reverse | GGTGAAGATTTCTCGATGGGC | 1400-1380 |
| *Ppia* | Peptidylprolyl isomerase A | NM_008907.1 | Forward | TGCTGGACCAAACACAAACG | 347-366 |
|  |  |  | Reverse | GCCTTCTTTCACCTTCCCAAA | 446-426 |
| *Ywhaz* | Tyrosine 3-monooxygenase/tryptophan 5-monooxygenase activation protein, zeta polypeptide |  | Forward | TCCTTATTCCCTCTTGGCAG | 2432–2451 |
|  |  |  | Reverse | ATGGAAGCTACATTAGCGGTTT | 2502–2523 |
| **Supplementary table 2. Primer information for RT-qPCR experiments.** | | | | | |
